# Characterizing user demographics in posts related to breast, lung and colon cancer on Japanese twitter (X)

**DOI:** 10.1038/s41598-024-56679-x

**Published:** 2024-03-18

**Authors:** Maho Kusudo, Mitsuo Terada, Nari Kureyama, Yumi Wanifuchi-Endo, Takashi Fujita, Tomoko Asano, Akiko Kato, Makiko Mori, Nanae Horisawa, Tatsuya Toyama

**Affiliations:** 1 Department of Breast Surgery, Nagoya City University Graduate School of Medical Sciences, 1 Kawasumi, Mizuho-cho, Mizuho-ku, Nagoya, Aichi 467-8601 Japan; 2 Department of Breast Oncology, Aichi Cancer Center Hospital, 1-1 Kanokoden, Chikusa-ku, Nagoya, Aichi 464-8681 Japan

**Keywords:** Breast cancer, Public health

## Abstract

Various cancer-related information is spreading on social media. Our study aimed to examine the account types associated with cancer-related tweets (currently known as posts) on Twitter (currently known as X) in Japan, specifically focusing on breast, lung, and colon cancer. Using the Twitter application programming interface, we collected tweets containing keywords of the three cancers type in August–September 2022. The accounts were categorized into seven types: Survivor, Patient’s family, Healthcare provider, Public organization, Private organization, News, and Other according to account name and texts. We analyzed the sources of the top 50 most liked and retweeted tweets. Out of 7753 identified tweets, breast cancer represented the majority (62.8%), followed by lung cancer (20.8%) and colon cancer (16.3%). Tweets came from 4976 accounts. Account types varied depending on the cancer type, with breast cancer topics more frequently from Survivor (16.0%) and lung cancer from Patient’s family (16.3%). Healthcare provider and Public organization had minimal representation across three cancer types. The trends in the top 50 tweets mirrored the distribution of accounts for each cancer type. Breast cancer-related tweets had the highest frequency. There were few from public organizations. These findings emphasize the need to consider the characteristics of cancer-related information sources when sharing and gathering information on social media.

## Introduction

Social media platforms have become an essential means for disseminating public health information and promoting health communication worldwide^1–3^. As of 2021, approximately 72% of adults in the United States and 84% of those between age 18 and 29 years reported using at least one social media platform^4,5^. Social media enables users to share public health messages and can potentially influence users’ health care outcomes^6^. Reportedly, 72% of adult Internet users have searched for health-related information online^7^. Additionally, among 1745 adults surveyed, 31.6% used social media to obtain health-related information^8^. Many potential benefits of social media use by patients with cancer have been reported. These include providing a platform for patient involvement and empowerment, enhancing both psychosocial and informational support, strengthen the relationship between patients and physicians, and offering avenues for participation in clinical and research studies^6^. However, potential drawbacks of social media have also been reported^6^, including misinformation^9,10^. We previously demonstrated that of the top 100 tweets with the most “likes” related to drug efficacy, side effects, and symptoms relevant in the cancer-related topic, 44% contained misinformation, 31% contained harmful information^10^. Although it is advisable to approach health information on social media with some level of skepticism and seek validation from medical professionals, it is equally crucial for public health organizations and specialized health care providers to disseminate reliable and accurate information. Public health organizations have widely adopted these platforms for communication purposes^3,11^. However, rates of social media adoption vary among countries^12^. In Japan, fewer hospitals and clinics use social media than in other countries; social media is likely to be used for public relations but not for providing medical information^13^.

It remains unclear how organizations, medical institutions, and healthcare providers are disseminating information and the promotion of awareness about cancer across various types of the disease in Japan.

Twitter (currently known as X) is one of the most popular social media platforms globally, with more than 330 million monthly active users worldwide^14^ and 59 million in Japan in 2023^15^.

Japanese patients with cancer also utilize Twitter as a communication tool in their patient journey^13,16^. In 2021, 38.5% of Twitter users globally were aged 25–34 years. The second-largest age demographic comprised users aged 35–49 years, accounting for nearly 21%; users aged 50 years and above accounted for 17%^17^. The use rate of social media also varies according to user age as well as country and region. The age of cancer onset varies contingent upon cancer type. Breast cancer has the highest incidence rates among women in their 40s and 60s in Japan, with a younger age of onset compared with other major cancer types and a lower age of onset for breast cancer than in other countries^18–21^. The susceptible age of onset of patients with breast cancer overlaps with the age groups that most often use social media, in comparison with lung cancer^22^ and colon cancer^23^.

On the basis of this background, we hypothesized that the types of users and the trends regarding dissemination of information differ according to cancer type. Comprehending the specific cancer types being addressed and identifying the types of accounts involved can yield insightful information. This knowledge is beneficial for the government, medical institutions/organization, and health care providers in formulating strategies for cancer awareness initiatives. Additionally, it serves as a crucial guide for patients and their families in navigating their interaction with this information. In this study, we investigated tweets (currently known as posts) related to breast, lung, and colon cancer on Twitter to reveal the demographics of users according to cancer type in Japan.

## Materials and methods

### Data acquisition and classifying Twitter accounts

Tweet data were retrospectively collected from August 19 to September 1, 2022 by querying the Twitter application programming interface (API) with the keywords “breast cancer,” “lung cancer,” and “colon cancer” in Japanese using Jupyter Notebook ver. 6.3.0^24^. Tweet data included the account name, tweet texts, profile description, number of followers, and the number of “likes” and “retweets (currently known as reposts).” We reviewed all user accounts and tweet texts and excluded retweets with no comments to identify the number of original tweets and accounts (original tweet data set) as they did not contain any specific opinions of the individuals who retweeted them. The number of accounts was calculated by excluding duplicated accounts from the original tweet data set. Each account was classified by at least two out of three investigators into the following seven categories: “Survivor,” “Survivor’s family,” “Health care provider,” “Public organization,” “Private organization,” “News,” and “Other,” according to the account name, description in the profile, and tweet texts. "Survivor" was defined as individuals who have undergone cancer treatment, and "Survivor's family" denoted family members of Survivors with cancer. "Health care provider" encompassed professional individuals like doctors, nurses, and other personnel working in health care settings. “Public organization” included entities like public hospitals, public research institutes, and governmental bodies, such as the Ministry of Health, Labour, and Welfare, as well as various academic societies. “Private organization” included private hospitals, health care provider or patient groups, and volunteer groups. "News" comprised different sources of news like TV, the Internet, and roundup websites that are websites compiling and presenting information on specific topics. "Other" comprised accounts that were ineligible for inclusion in the other six categories including accounts such as anonymous accounts that lacked of sufficient information for categorization.

### Outcomes

To characterize the demographic profile of accounts and tweets mentioning the above three major cancers in Japan, we initially calculated the number of accounts, excluding duplicated accounts, from the original tweet data set. We documented the proportion of account categories, as well as the overall number of tweets, for each cancer type. We compared the number of followers across account categories and cancer types to identify trends among influencers and high-profile accounts for each cancer type. We examined accounts with the 50 tweets that had the most likes and retweets.

### Statistical analysis

We performed the chi-square test to determine statistical differences in the proportion of account categories for each cancer type. We performed analysis of variance (ANOVA) to compare the number of tweets and followers, adjusted using Dunnett’s multiple comparison test. We considered p values < 0.05 to be statistically significant. All analyses were conducted using GraphPad Prism ver. 9.0.0 (GraphPad LLC, San Diego, CA, USA).

### Ethics approval and informed consent

The protocol for this study (no. 60-22-0148) was approved by the institutional review board of Nagoya City University Graduate School of Medical Sciences in April 2023. All human research was conducted according to the Declaration of Helsinki and Twitter’s Developer Agreement and Policy. The institutional review board of Nagoya City University Graduate School of Medical Sciences stated to waive the need for informed consent requirement in this study. Our study received approval from Twitter on Aug 2022, granting us permission to utilize the academic level of Twitter API.

## Results

### Overview of cancer-related tweets and accounts

**Figure 1:**
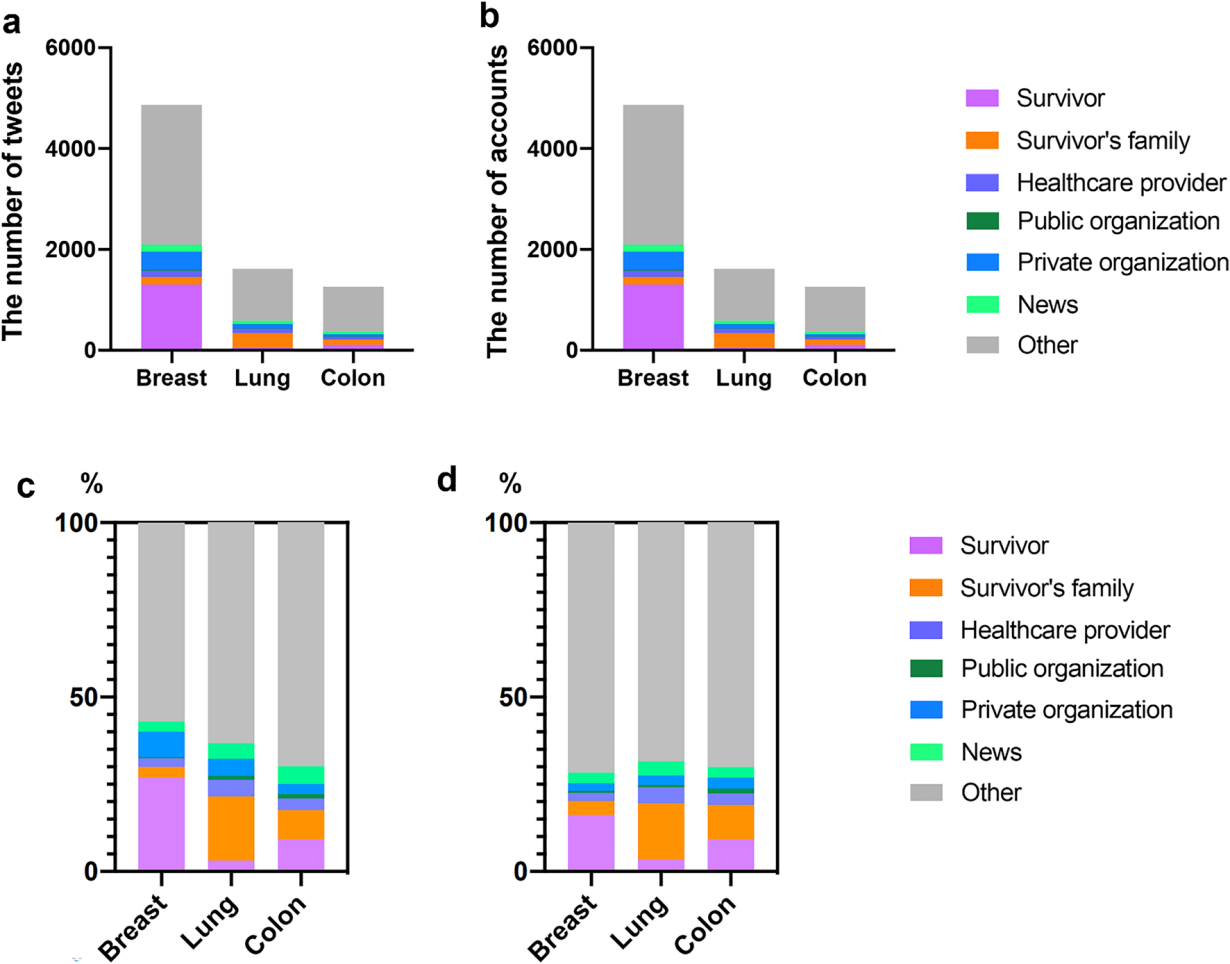
Total number of tweets and accounts and the proportion of account categories. Comparison of the total number of (**a**) tweets and (**b**) accounts, by cancer type. Proportion of account categories by (**c**) number of tweets, and (**d**) number of accounts.

Between August 19, 2022 and September 1, 2022, we identified a total of 16,355 tweets related to breast cancer, 3791 related to lung cancer, and 3033 related to colon cancer. After excluding retweets without any comments, the total number of original tweets related to breast cancer, lung cancer, and colon cancer was 4871, 1616, and 1266, respectively (Fig. 1a). The number of accounts related to breast cancer, lung cancer, and colon cancer was 3086, 1002 and 888, respectively (Fig. 1b). We categorized the accounts into seven categories for each cancer type. The proportions of account categories for each cancer type are shown according to the number of tweets in Fig. 1c and by the number of accounts in Fig. 1d. Breast cancer accounts were classified as follows: Survivor, 16.0% (n = 494); Survivor’s family, 4.1% (n = 126); Health care provider, 2.5% (n = 76); Public organization, 0.5% (n = 15); Private organization, 2.3% (n = 70); News, 3.0% (n = 94); and Other, 71.6% (n = 2211). Lung cancer accounts were classified as follows: Survivor, 3.4% (n = 34); Survivor’s family, 16.3% (n = 163); Health care provider, 4.4% (n = 44); Public organization, 0.7% (n = 7); Private organization, 2.7% (n = 27); News, 4.2% (n = 42); and Other, 68.4% (n = 685). Colon cancer accounts were classified as follows: Survivor, 9.1% (n = 81); Survivor’s family, 10.0% (n = 89); Health care provider, 3.2% (n = 28); Public organization, 15% (n = 13); Private organization, 3.0% (n = 27); News, 3.2% (n = 28); and Other, 70.0% (n = 622). The composition of accounts in the total number of accounts was significantly different for each cancer type (p < 0.0001). The category of Other accounted for the largest proportion of all cancer types. Accounts posting breast cancer-related tweets had a higher proportion of the Survivor category than other cancer types, and accounts posting lung cancer-related tweets included more of the Survivor’s family category than other cancers. The proportions of Health care provider and Public organization accounts were small for each cancer type. The number of original tweets by account category for each cancer type showed a similar trend in the composition of accounts for each cancer type.

### Follower analysis

**Figure 2:**
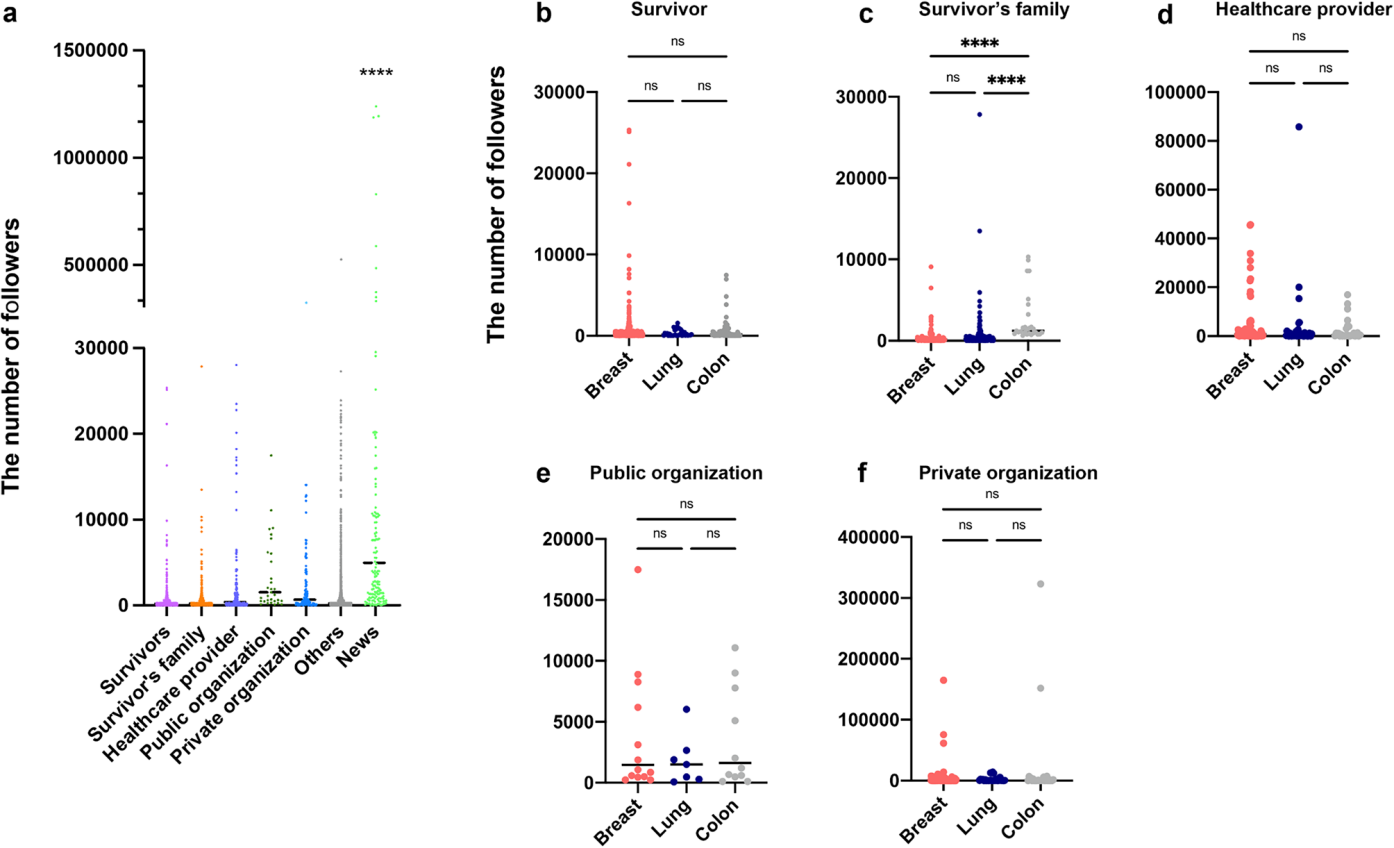
Comparison of number of followers. The total number of followers by account category in all cancer types (**a**). The number of followers of (**b**) Survivor, (**c**) Survivor’s family, (**d**) Health care provider, (**e**) Public organization, and (**f**) Private organization accounts for each cancer type. Analysis of variance was performed to compare the number of tweets and followers, adjusted with Dunnett’s multiple comparison test. p < 0.05 was considered significant (ns, not significant. ****p < 0.0001).

We compared the number of followers across the various account categories and cancer types. In all cohorts including breast, lung, and colon cancer, News had the largest number of followers among the seven categories. News had significantly more followers (p < 0.0001), but there were no differences among the other six account categories in the adjusted multiple comparison test (Fig. 2a). The number of followers in the categories Survivor, Public organization, and Private organization were comparable among each cancer type (Fig. 2b–f). Survivor’s family had significantly more followers for colon cancer than breast and lung cancer (p < 0.0001, Fig. 2c).

### Analysis of top 50 tweets

**Figure 3:**
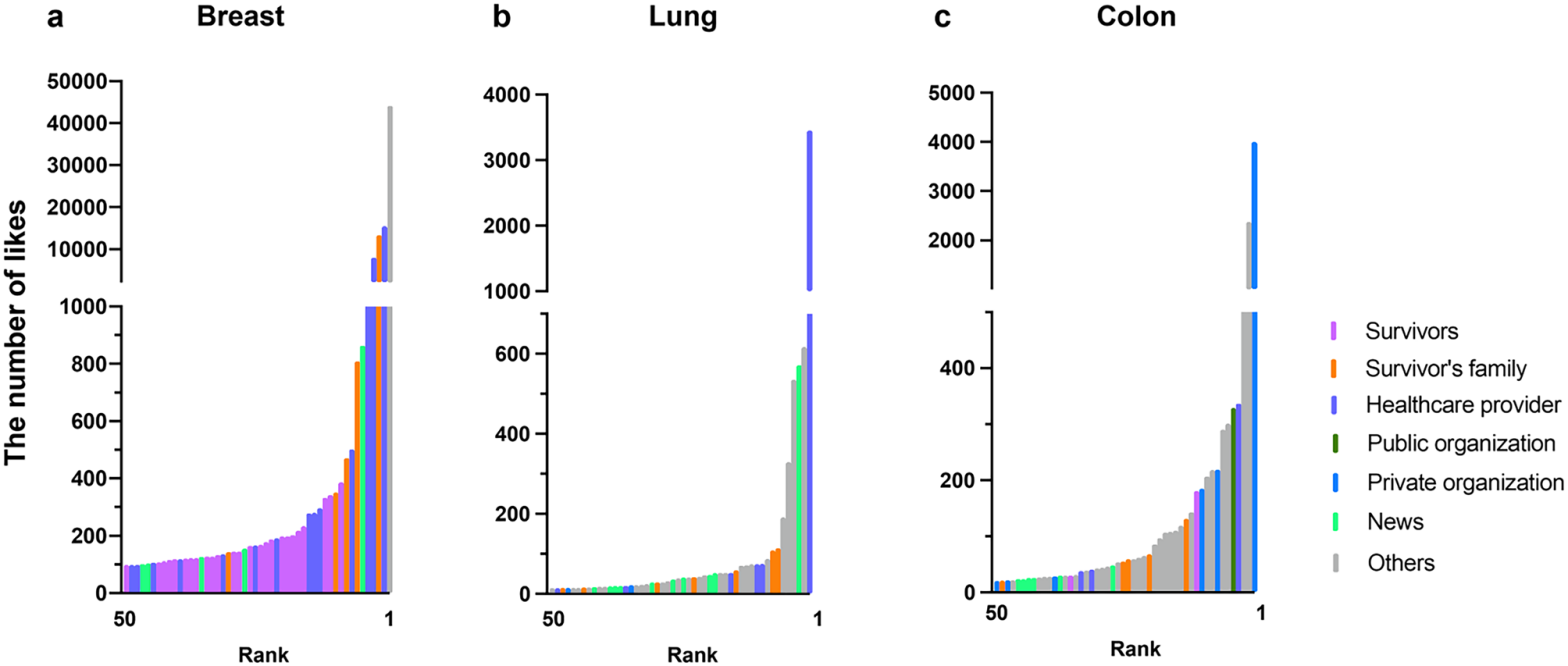
Account category trends in the leading 50 liked tweets. The number of likes for the top 50 tweets with each account shown in a different color in (**a**) breast, (**b**) lung, and (**c**) colon cancer.

We sorted and analyzed the leading 50 tweets in terms of the number of likes and retweets, according to each cancer type; a summary is shown in Table 1. Among the top 50 likes, posts from Survivor accounts were the most frequent (50.0%) in breast cancer (Fig. 3a); in lung cancer (Fig. 3b), Other (50.0%) accounts were the most common, followed by News (20.0%), Survivor’s family (14.0%), and Health care provider (12.0%) accounts. In colon cancer (Fig. 3c), Other accounts were the most frequent (54.0%), followed by Private organization (12.0%), News (12.0%), and Survivor’s family (10.0%) accounts. In all three types of cancer, tweets from Public organization accounts were nearly absent from the ranking (0.0%, 0.0%, and 2.0% respectively). Among the leading 50 retweets, News and Other accounts were most prevalent for breast (Supplementary Fig. 1a), lung (Supplementary Fig. 1b), and colon cancers (Supplementary Fig. 1c). The proportion of posts among the top 50 tweets from Public organization accounts was 2.0% for breast cancer, 0.0% lung cancer, and 8.0% for colon cancer.

**Table 1:** Account classification of leading 50 liked and retweeted tweets.

	Breast		Lung		Colon	
	n	%	n	%	n	%
Top 50 likes						
Survivors	25	50.0	0	0.0	2	4.0
Patient's family	1	2.0	7	14.0	5	10.0
Healthcare providers	5	10.0	6	12.0	3	6.0
Public organizations	0	0.0	0	0.0	1	2.0
Private organizations	0	0.0	2	4.0	6	12.0
Other accounts	14	28.0	25	50.0	27	54.0
News	5	10.0	10	20.0	6	12.0
Top 50 retweets						
Survivors	6	12.0	0	0.0	1	2.0
Patient's family	2	4.0	6	12.0	3	6.0
Healthcare providers	5	10.0	3	6.0	3	6.0
Public organizations	1	2.0	0	0.0	4	8.0
Private organizations	2	4.0	2	4.0	7	14.0
Other accounts	17	34.0	28	56.0	26	52.0
News	17	34.0	11	22.0	6	12.0

## Discussion

In this study, we aimed to reveal the characteristics of accounts posting cancer-related information on the Twitter social media platform. This was the first study to describe the characteristics of major cancer-related tweets in Japan. Among three types of cancer investigated, the most tweets were related to breast cancer. For three types of cancer, a relatively low proportion of tweets originated from health care providers or public organizations. However, user backgrounds varied depending on the type of cancer, with breast cancer topics mainly tweeted by breast cancer survivors and lung cancer topics mostly tweeted by Survivor’s family members. Our study suggested that the user demographics and purposes of using social media differ depending on the type of cancer. We also analyzed which account types were most likely to have an impact, finding that tweets from health care providers or public institutions did not rank highly. We found that tweets from news accounts were likely to receive more attention, and for breast cancer in particular, survivors were highly represented among the top 50 most liked tweets.

According to a survey by Israeli Stop Cancer, an online social media platform with more than 20,000 registered members, the most common diagnoses were breast cancer (31%), lymphoma (24%), leukemia (10%), and colon cancer (8%)^25,26^. Consistent with these previous reports, we demonstrated that breast cancer was the most prevalent type of cancer, followed by lung and colon cancers. The age of onset of the disease is believed to be a crucial factor contributing to the high number of tweets related to breast cancer. Compared with lung and colon cancer, patients with breast cancer include a higher proportion of adolescents and young adults (AYAs). AYAs undergoing cancer treatment tend to use social media platforms in distinctive ways. Social media can potentially facilitate social support for AYAs, thereby helping them overcome the challenges posed by traditional in-person support groups^6,25^. Additionally, social media can aid in connecting patients with similar diagnoses or in maintaining friendships formed during the course of treatment^6^. According to patient-reported outcomes in a Twitter survey for patients with breast cancer, participation in the Twitter #BCSM (Breast Cancer Social Media Twitter support community) reduced perceived anxiety related to breast cancer^27^. Some AYAs with cancer have reported the establishment of new and strong connections with peers met through social media platforms, leading to a less stigmatizing form of support.

Caregivers are acknowledged as a susceptible group that offers vital psychosocial assistance to patients with cancer, but caregivers are at higher risk of elevated psychological distress and unaddressed needs^28^. In particular, for pediatric cancer, creating a network of support on social media has been reported to assist parents in accepting their child’s diagnosis and coping with the situation^29^. Although we did not focus on pediatric cancer in our study, the proportion of accounts held by Survivor’ family members varied according to the type of cancer, with relatively high numbers observed for lung cancer related tweets. The degree of symptoms endured by patients with lung cancer has been documented to be more severe than those of other cancer types^30^, leading to compromised functionality with an accompanying detrimental effect on overall quality of life^31^. A systematic review reported that stress among caregivers of patients with lung cancer is influenced by factors such as the patient’s disease stage, the relationship with the patient, social support, and coping methods^28^. Given these backgrounds, our results suggest that families of patients with lung cancer may be more likely to seek support networks on social media.

A previous survey reported that the motivation among patients with cancer for using social media was for emotional coping in 74% of patients, and social media was used to obtain medical information in 45% of patients^25^. However, current regulations regarding the dissemination of such information are deemed inadequate. Patients and their family members must take responsibility for selecting the information to focus on, which can be challenging with a non-professional background. Because information that is accessible through social media can considerably affect the decisions made by patients, providing medically accurate and helpful information via social media is crucial. The dissemination of evidence-based information by governments, medical institutions, and professionals is of great importance. However, our study indicated that there were few cancer-related messages on Twitter from public institutions and health care providers, and these messages did not receive much attention. In Japan, hospitals and clinics are unlikely to use social media platforms like Twitter for health promotion purposes, and more than half of these institutions only post notifications or hospital and clinic news^13^. Although we did not analyze the specific characteristics of tweet content, our findings indicated that health-related information from public organizations on Twitter in Japan is limited. Social media platforms offer a valuable opportunity for oncology professionals to disseminate reliable and evidence-based information, making it a vital teaching platform. Physicians using social media can be classified into three groups: those involved in professional education or continuing professional development, public health messaging or education, and direct interaction with individual patients for clinical purposes^32^. By leveraging social media, awareness about clinical trials in oncology can be enhanced and participation rates can be increased. Furthermore, social media platforms can be used by physicians to distribute and exchange information with other health care professionals, making it an invaluable tool. However, our data indicate that health care providers and public organizations have not been very active in disseminating information about cancer, suggesting that medical professionals in Japan may not be effectively using social media to improve the informational environment surrounding cancer.

In the evaluation of follower count among the top 50 accounts, News accounts had a high number of followers and accounted for a considerable proportion of retweets and likes. This observation suggests that news accounts possess the ability to exert a substantial influence on a large number of people. A study of frequently accessed news articles about breast cancer indicated that 13% of news items, which were classified as rumors with low confidence levels, were shared 3.29 times more often than news with verified content^33^. The implications of that study are far-reaching as news stories that were classified as rumors were reportedly shared over 5,700,000 times. There were no dissimilarities in the quantity of followers across cancer types within Survivor, Health care provider, Public organization, and Private institution account types. However, the number of followers among Survivor’s family accounts was significantly greater for colon cancer, although the rationale behind this discrepancy is unclear. These findings indicate that the dissemination capacity according to account category did not vary significantly across the various types of cancer investigated.

This study had some limitations. First, the data collection period was short and limited because the regulations of the Twitter API only allowed us to search up to the 7 days prior to the search date. The implication of this study could change and may be different from time to time. Although it is unclear whether the distribution of accounts throughout the year was the same, our data collection date was chosen randomly, and while there was a risk that certain events might influence the content of tweets, it turned out that the search period did not coincide with any cancer-related academic conferences or international awareness events; therefore, the effect of the limited search period is likely to be small. Second, our cohort included a significant number of the Other accounts, as over 70% of Twitter users in Japan are anonymous^34^. The Other account group may have potentially included accounts that were classified incorrectly because we manually classified the account categories based on the user name or texts in profiles and posted tweets. Third, there may be accounts who engaged in discussions on the relevant cancer topics without utilizing the keywords employed in this study. To address this, further analysis using comprehensive language models akin to artificial intelligence is needed.

## Conclusion

Our study revealed the trends among senders of cancer-related tweets on the Twitter platform in Japan. Our findings highlight the potential benefits and harm of social media, depending on its use. The dissemination of accurate information is crucial for improving the safety of medical information shared on social media. Our study demonstrated that of the three major cancers investigated, the most popular topic of conversation on Japanese Twitter was related to breast cancer. Additionally, tweets from public institutions and health care providers were not highly represented, and the demographics of account holders varied depending on the type of cancer. We believe these data are crucial for health care providers to consider when planning future awareness campaigns. Notably, user backgrounds may vary depending on the study period and social media platform; further research in this area is necessary. Developing awareness strategies adapted to the nature of social media platforms in Japan is also crucial to consider.

### Supplementary Information

Supplementary Legends.

Supplementary Figure 1.

## Data Availability

The datasets used and analyzed during the current study are available from the corresponding author on reasonable request.

## Abbreviations

API: Application programming interface
ANOVA: Analysis of variance
AYAs: Adolescents and young adults
BCSM: Breast Cancer Social Media Twitter support community
