# Supplementary material for: Characterizing user demographics in posts related to breast, lung and colon cancer on Japanese twitter (X)
Source: Sci Rep. 2024 Mar 18;14:6485. doi: 10.1038/s41598-024-56679-x (PMC10948868; doi:10.1038/s41598-024-56679-x)
Supplement: Supplementary file 1 — Supplementary Legends. [file 41598_2024_56679_MOESM1_ESM.docx]

**Supplementary Figure Legend**

**Supplementary Fig. 1 Account category trends in the leading 50 retweeted tweets**

The number of retweets for the top 50 tweets with each account shown in a different color in a) breast, b) lung, and c) colon cancer.
