# Supplementary figures and images for: Characterizing user demographics in posts related to breast, lung and colon cancer on Japanese twitter (X)
Source: Sci Rep. 2024 Mar 18;14:6485. doi: 10.1038/s41598-024-56679-x (PMC10948868; doi:10.1038/s41598-024-56679-x)

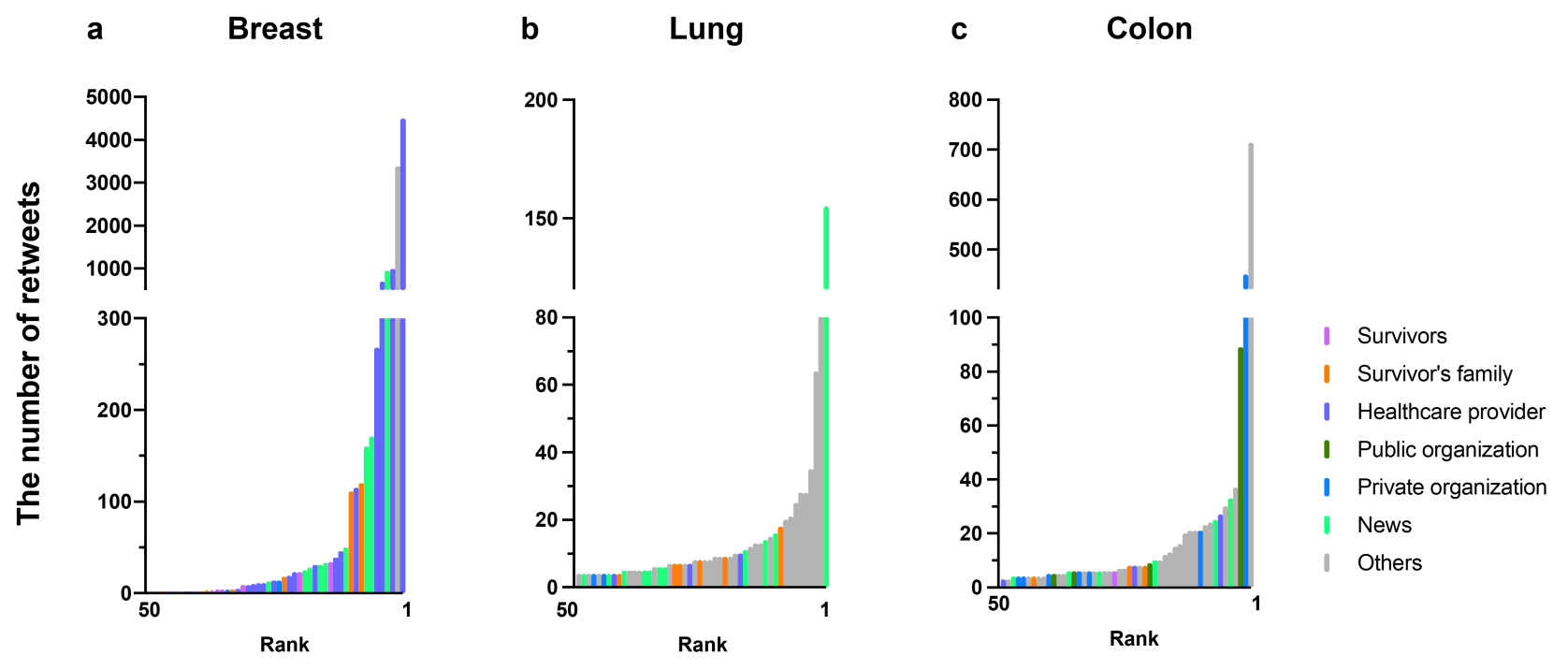

Supplement: Supplementary file 2 — Supplementary Figure 1. [file 41598_2024_56679_MOESM2_ESM.tif]
